# Are YouTube Videos a Reliable Training Method for Safe Laparoscopic Cholecystectomy? A Simulated Decision-Making Exercise to Assess the Critical View of Safety

**DOI:** 10.1055/s-0041-1740627

**Published:** 2021-12-23

**Authors:** Dimitrios K. Manatakis, Emmanouil Mylonakis, Petros Anagnostopoulos, Konstantinos Lamprakakis, Christos Agalianos, Dimitrios P. Korkolis, Christos Dervenis

**Affiliations:** 1 2nd Department of Surgery, Athens Naval and Veterans Hospital, Athens, Greece; 2 Department of Surgical Oncology, Saint Savvas Cancer Hospital, Athens, Greece; 3 Department of Surgery, Naval Hospital of Crete, Chania, Greece; 4 Department of Surgery, Medical School, University of Cyprus, Nicosia, Cyprus

**Keywords:** surgical training, laparoscopic cholecystectomy, bile duct, critical view of safety (CVS).

## Abstract

**Background** The present study assesses the educational value of laparoscopic cholecystectomy videos on YouTube regarding the correct application of the critical view of safety (CVS), and evaluates… surgical trainees' perceptions of the CVS criteria in a simulated, operative decision-making exercise.

**Methods** YouTube was systematically searched for laparoscopic cholecystectomy videos, explicitly reporting a satisfactory CVS. The top 30 most popular videos, by number of views, were identified and scored on the 6-point scale by three experienced consultants. After watching a training module on CVS rationale and criteria, 10 trainees, blinded to the consultants' assessment, were instructed to view the videos, score each criterion and answer the binary question “Would you divide the cystic structures?” by “yes” or “no.”

**Results** An inadequate CVS was found in 30% of the included videos. No statistical association was noted between number of views, likes, or dislikes with successful CVS rates. Inter-observer agreement between consultants and trainees ranged from minimal to moderate ( *k* = 0.07–0.60). Discrepancy between trainees' CVS scores and their simulated decision to proceed to division of the cystic structures was found in 15% of assessments, with intra-observer agreement ranging from minimal to excellent ( *k* = 0.27–1.0). For the CVS requirements, inter-observer agreement was minimal for the dissection of the cystic plate ( *k* = 0.26) and triangle clearance ( *k* = 0.39) and moderate for the identification of two and only two structures ( *k* = 0.42).

**Conclusion** The CVS is central to the culture of safety in laparoscopic cholecystectomy. Surgical videos are a useful training tool as simulated, operative decision-making exercises. However, public video platforms should be used judiciously, since their content is not peer-reviewed or quality-controlled.

Technological advances and web-based applications have truly revolutionized the field of surgical education. In an effort to enhance the learning experience, traditional tools, like the time-honored atlas of surgical operations, have given way to contemporary solutions, like simulators and virtual reality. 1 Undoubtedly, the audiovisual interaction offered by modern technology cannot be surpassed by books and amphitheater lectures.

Surgical videos are an example of the benefits of multimedia in the learning process and have been established as a modern educational module. Ninety percent of surgeons and trainees watch videos to prepare for surgical cases and studying, usually on public platforms like YouTube ( www.youtube.com ). 2 3 4 However, the true value of public video libraries, as source of medical information or surgical technique, has been questioned, due to concerns for low quality, non—peer-reviewed material, or nonadherence to guidelines. 5 6 7 8 9 10

To date, only a small number of studies have evaluated the quality of laparoscopic cholecystectomy videos on popular platforms (YouTube, Vimeo, etc.), with regard to the CVS principles. 1 11 12 13 14 They have found alarmingly low rates of correct CVS application, as low as 10 to 28%. However, selection of videos in these studies was based on the search term “laparoscopic cholecystectomy” alone, without any reference to the CVS itself.

The aim of our study was to assess the educational value of laparoscopic cholecystectomy videos on YouTube, that explicitly reported successful CVS achievement. These videos were used for a simulated decision-making exercise, aimed at evaluating surgical trainees' perceptions of the CVS criteria.

## Materials and Methods

Introduced in 1995, the CVS is a method of target identification used in laparoscopic cholecystectomy, to avoid bile duct and vascular injuries, due to misidentification of anatomical structures in the hepatocystic triangle. 15 Secure identification of the cystic structures depends on three requirements: (1) clearance of the hepatocystic triangle of all fibrofatty tissue, (2) identification of two and only two tubular structures entering the gallbladder wall, and (3) dissection of the lower third of the gallbladder off the cystic plate. Each criterion is awarded 0 to 2 points, for a maximum of six points. 16 Scores of 5 or 6 are considered a satisfactory CVS, allowing safe ligation of the cystic duct and artery. Scores of 0 to 4 require further dissection or a bail-out technique. 17

Using the keywords “critical view of safety” and “laparoscopic cholecystectomy,” the YouTube platform was searched on May 21 ^st^ , 2020, to identify operative videos, which explicitly reported a satisfactory CVS, either in the title, the description, or embedded on the video itself. Eligible were videos of live surgical procedures with adequate play time to enable assessment of the dissection of the hepatocystic triangle and demonstration of the CVS criteria. Animations, lectures, conference presentations, and educational material provided by scientific societies were excluded. No restrictions on age, gender, ethnicity, or experience of the primary surgeon were imposed. Both videos of elective and acute cholecystitis cases were acceptable. For each video, the following data were extracted: URL, number of views, likes, dislikes and comments, gender, and country of the primary surgeon.

Three consultant surgeons, trained on and exclusively performing the CVS approach, jointly scored the videos, using the six-point scale. Videos were judged as a whole, therefore, points were given if the relevant CVS requirement was present either in the anterior or the posterior view. Each video was subsequently characterized as either “Pass” (5–6) or “Fail” (0–4). A subanalysis was performed, to assess whether number of views, likes and dislikes were associated with rates of successful CVS.

The video URLs were then given to 10 trainees [five junior (years 1–3) and five senior (years 4–5) residents], blinded to the consultants' assessment. After watching a training module on CVS rationale and criteria with operative examples, they were instructed to view the videos, until the cystic structures were clipped and divided, to score each CVS criterion and answer the binary question “ *Would* you *divide the cystic structures?* ” by “Yes” or “No.”

Ιnter-observer agreement was calculated for each resident as percent agreement and Randolph's kappa, by comparison with consultants' score. 18 Intra-observer agreement among trainees was also calculated, comparing their CVS evaluation and decision to divide or not the cystic structures. To further determine which CVS criterion was the most difficult to identify, the gradings for each criterion were compared among residents by Randolph's kappa. Statistical analysis was performed on SPSS ver 20.0 (IBM Corp.). Values of kappa coefficient were interpreted as follows: no agreement (0–0.20), minimal agreement (0.21–0.40), weak agreement (0.41–0.60), moderate agreement (0.61–0.80), strong agreement (0.81–0.90), and almost perfect agreement (>0.90).

## Results

The 30 highest ranking videos, by number of views, were included in the study ( Table 1 ). Median number of views was 2,313 (range 331–58,541). All surgeons were males.

**Table 1:** Characteristics of included YouTube videos

No	Views	Likes	Dislikes	Comments	Surgeon gender	Country	Setting
1	58,541	368	46	64	Male	India	Elective
2	33,183	90	19	8	Male	United States	Elective
3	28,374	78	4	3	Male	Egypt	Elective
4	28,311	141	8	3	Male	India	Elective
5	10,171	46	6	15	Male	Turkey	Acute
6	9,657	35	2	5	Male	Turkey	Elective
7	7,800	54	6	0	Male	Egypt	Elective
8	6,049	29	7	4	Male	Italy	Elective
9	4,424	24	2	2	Male	Italy	Acute
10	3,257	6	0	3	Male	India	Elective
11	3,181	24	2	4	Male	Turkey	Acute
12	2,951	8	2	0	Male	UK	Elective
13	2,409	23	5	5	Male	India	Elective
14	2,408	18	1	1	Male	United States	Elective
15	2,374	51	1	4	Male	Argentina	Elective
16	2,251	28	0	3	Male	Argentina	Elective
17	1,914	11	2	0	Male	Turkey	Elective
18	1,515	17	0	5	Male	Italy	Acute
19	1,324	4	0	0	Male	India	Elective
20	1,080	1	2	0	Male	India	Elective
21	980	26	0	11	Male	India	Elective
22	925	32	2	6	Male	Italy	Elective
23	888	5	0	0	Male	Greece	Elective
24	885	32	1	4	Male	India	Elective
25	864	12	0	1	Male	United States	Elective
26	805	24	0	0	Male	Argentina	Elective
27	604	5	0	2	Male	Turkey	Acute
28	516	5	0	1	Male	Turkey	Elective
29	344	2	1	0	Male	India	Acute
30	331	2	0	0	Male	United States	Elective

Twenty-one videos (70%) were judged by the consultants as having properly obtained the CVS (scores of 5–6), whereas 9 (30%) were deemed unsatisfactory (scores of 0–4). Out of 24 elective and six acute cholecystitis cases, the CVS was not obtained in 5 (20.8%) and 4 (66.7%), respectively. No statistical association was observed between number of views, likes or dislikes with completion rates of CVS.

**Fig. 1:**
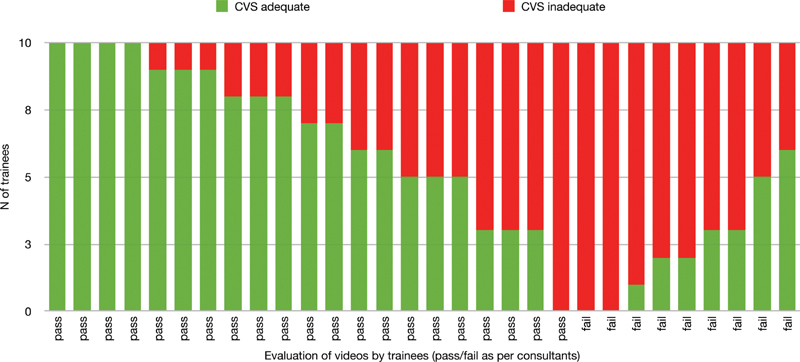
Summary of trainees' assessment of the top-30 YouTube CVS videos [pass = adequate CVS (5–6), fail = inadequate CVS (0–4); as judged by consultants]. CVS, critical view of safety.

**Fig. 2:**
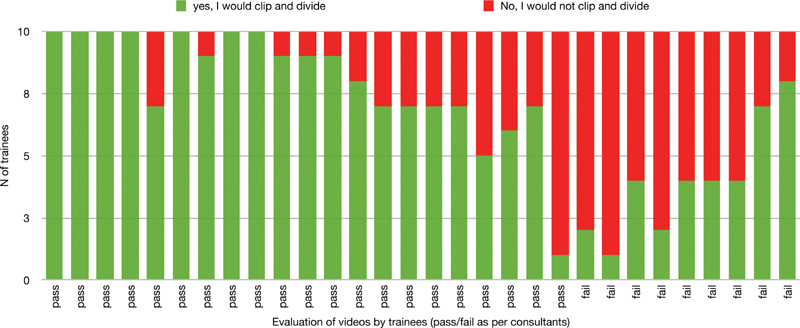
Summary of trainees' responses to the binary question “Would you divide the cystic structures?” [pass = adequate CVS (5–6), fail = inadequate CVS (0–4); as judged by consultants]. CVS, critical view of safety.

Table 2 shows the trainees' evaluation. Overall “Pass” ratings ranged between 30 and 76.7%, while decision to proceed with division of the cystic structures ranged between 53.3 and 83.3% (discrepancy in 45/300 assessments). The inter-observer agreement between consultants and residents is shown in Table 2 and Fig. 1 . Percent agreement ranged between 53 and 80% (mean 69.7 ± 9.2%), whereas Randolph's kappa between 0.07 and 0.60 (mean 0.39 ± 0.18). Intra-observer agreement between the trainees' CVS scoring and the decision to divide the structures ranged between 63.3 and 100% (as percent agreement) and 0.27 to 1 (as Randolph's kappa). ( Fig. 2 ). Level of trainee surgical experience was not associated with higher levels of inter- and intra-observer agreement. For the three CVS requirements, inter-observer agreement was minimal for dissection of the cystic plate ( *k* = 0.26) and triangle clearance ( *k* = 0.39) and weak for the identification of 2 structures ( *k* = 0.42).

**Table 2:** Inter- and intraobserver agreement between consultants and trainees

	**Experience**	**Pass (5–6)** ***n*** **(%)**	**Fail (0–4)** ***n*** **(%)**	**% Agreement**	**Inter-obsever agreement (kappa)**	**Would you clip? yes (%)**	**% Agreement**	**Intra-observer agreement (kappa)**
Consultants		**21 (70)**	**9 (30)**					
Trainee no 1	Senior	10 (33.3)	20 (66.7)	63.3%	0.27	21 (70)	63.3%	0.27
Trainee no 2	Senior	15 (50)	15 (50)	80%	0.60	16 (53.3)	96.7%	0.93
Trainee no 3	Senior	19 (63.3)	11 (36.7)	60%	0.20	19 (63.3)	93.3%	0.87
Trainee no 4	Senior	18 (60)	12 (40)	70%	0.40	18 (60)	100%	1
Trainee no 5	Senior	17 (56.7)	13 (43.3)	80%	0.60	25 (83.3)	73.3%	0.47
Trainee no 6	Junior	16 (53.3)	14 (46.7)	76.7%	0.53	19 (63.3)	90%	0.80
Trainee no 7	Junior	18 (60)	12 (40)	76.7%	0.53	18 (60)	100%	1
Trainee no 8	Junior	23 (76.7)	7 (23.3)	73.3%	0.47	23 (76.7)	93.3%	0.87
Trainee no 9	Junior	18 (60)	12 (40)	63.3%	0.27	25 (83.3)	76.7%	0.53
Trainee no 10	Junior	9 (30)	21 (70)	53.3%	0.07	20 (66.7)	63.3%	0.27

## Discussion

Previous studies assessing laparoscopic cholecystectomy videos on internet platforms found disappointing rates of proper CVS application. Lee et al analyzed 73 YouTube videos, grading 15% as “good,” 55% as “moderate” and 30% as “poor.” 11 However, demonstration of the CVS requirements was not reported by the 6-point scale, but rather on a 0 to 3 scale, and was incorporated into the overall scoring. Deal et al reviewed 40 representative videos on YouTube, Vimeo and the SAGES library, of variable technical performance. 13 Faculty expert ratings showed that only 12.5% achieved CVS scores of ≥5, while 85% scored ≤3. Moreover, an analysis of 139 YouTube videos by the same team, using crowd-sourcing, found no statistical correlation between number of views, likes, dislikes or subscribers and the completion of CVS. 12 Rodriguez et al analyzed the top ten listed YouTube videos and found only one to show a satisfactory CVS (≥5), while Chavira et al reviewed a total of 77 YouTube, WebSurg and GIBLIB videos, showing successful CVS rates in 27.7, 44.4, and 40%, respectively. 1 14

The aforementioned studies used the search term “laparoscopic cholecystectomy” during the selection process. By combining the keywords “critical view of safety” *AND* “laparoscopic cholecystectomy,” our analysis included only those videos that explicitly reported the successful application of the CVS. Of the top-30 most popular videos by number of views, 70% were evaluated by consultants as having conclusively achieved a CVS score of 5 or 6. Expectedly, this percentage was higher compared with similar studies of unselected cases. However, our results showed that a significant proportion of surgeons (30%), although claiming a satisfactory CVS, still misunderstood the core concept in practice.

In addition, similar to previous studies, the number of views, likes, and dislikes was not associated with successful CVS rates. 1 12 Indeed, the significant difference in views between the first and last video (58,541 vs. 331) shows that viewers rarely scroll beyond the first few results. 14 Even more importantly, neither likes nor dislikes reflect the actual quality of the content. Public video libraries should therefore be used very judiciously, since the uploaded content is usually not peer-reviewed. Dedicated surgical websites and operative videos produced by academic institutions or official surgical societies are of higher educational value and should be preferred as training material.

The simulated decision-making exercise revealed certain interesting results. Between trainees and consultants, inter-observer agreement ranged from minimal to moderate. Generally, trainees tended to give lower marks overall, compared with consultants. To some extent, this fact may be attributed to a more cautious evaluation of the CVS criteria by surgeons in the beginnings of their learning curve. On the other hand, higher level of surgical training was not associated with higher inter-observer agreement rates, as might have been expected.

Yet, even more contradictory was the discordance between CVS scores and decision to divide the cystic structures. Despite the explanatory training module prior to the exercise, in 15% of cases the trainees would indeed proceed to ligation, even though their awarded CVS score was <5. This misconception of the CVS rationale is a hazardous gap in surgical training and could ultimately reflect an unsafe practice.

While the learning curve for mastering the CVS has yet to be determined, by analysis and comparison of operative notes and videos we do know that even experienced surgeons may lack full understanding of the three steps that constitute a proper CVS. 19 20 Given the burden of BDI on the health care system and patients' long-term quality of life, education of surgeons toward the correct application of the CVS cannot be overemphasized. 19 21 Tutorials with structured curriculum are necessary to highlight the rationale behind the CVS requirements and promote a culture of safety in laparoscopic cholecystectomy. Either in the form of lecture or video, they have been shown to increase rates of successful CVS and improve confidence among trainees. 21 22 23 24 25 26

Furthermore, we found considerable variation in the evaluation of each CVS criterion, with least agreement for adequate dissection of the cystic plate, similar to Mascagni et al. 27 Mobilization of the lower third of the gallbladder off the liver bed is essential, to secure that the purported cystic structures do not reenter the hepatic parenchyma at a higher level. However, there is not one single reason why surgeons fail to obtain a proper CVS. Nakazato et al found that the most common cause for an incomplete CVS was the inadequate cystic plate dissection, Carr et al found the inadequate clearance of the hepatocystic triangle, while Nijssen et al found the inability to recognize two and only two structures. 20 25 26 Equal emphasis on all three requirements is therefore necessary.

Inconsistency and subjective interpretation of the CVS criteria hide a dangerous trap, that could lead to vasculobiliary injuries. A conceptual framework, developed by expert academic surgeons, defined the essential competencies required to establish the proper CVS. 28 This framework includes cognitive elements and potential errors, related to situational awareness, decision-making and action-oriented subtasks, and may serve as the basis for surgical training, assessment tools, and quality-control metrics.

Our study was limited by the small number of participants, all of them trainees at a single surgical department, as well as small number of videos ( *n* = 30). We also narrowed the selection of surgical videos to YouTube and chose not to include specialized, online surgical libraries. The videos were included solely on the basis of popularity and were not assessed for their technical quality or surgical competency, thus better reflecting real-world situations. Nevertheless, they were evaluated by experienced consultants using the recommended six-point CVS scale. Finally, our training module was similar in concept to the video tutorial by Deal et al, but has not been validated as an educational tool. 24

## Conclusion

Promotion of a culture of safety should be the very core of laparoscopic cholecystectomy training. And the CVS concept is central to this culture. Surgical videos are a useful educational tool, as simulated decision-making exercises. However, public video platforms should be used judiciously by trainees, since their content is not peer-reviewed or quality-controlled.
